# A Phylogenetic Perspective on the Individual Species-Area Relationship in Temperate and Tropical Tree Communities

**DOI:** 10.1371/journal.pone.0063192

**Published:** 2013-05-01

**Authors:** Jie Yang, Nathan G. Swenson, Min Cao, George B. Chuyong, Corneille E. N. Ewango, Robert Howe, David Kenfack, Duncan Thomas, Amy Wolf, Luxiang Lin

**Affiliations:** 1 Key Laboratory of Tropical Forest Ecology, Xishuangbanna Tropical Botanical Garden, Chinese Academy of Sciences, Kunming, China; 2 Graduate University of Chinese Academy of Sciences, Beijing, China; 3 Department of Plant Biology, Michigan State University, East Lansing, Michigan, United States of America; 4 Department of Botany and Plant Physiology, University of Buea, Buea, Cameroon; 5 Centre de Formation et de Recherche en Conservation Forestière (CEFRECOF) Epulu, Ituri Forest, Reserve de Faune a Okapis, Democratic Republic of Congo; 6 Department of Natural and Applied Science, University of Wisconsin – Green Bay, Green Bay, Wisconsin, United States of America; 7 Smithsonian Global Earth Observatory Network, Smithsonian Institution, Washington, D.C., United States of America; 8 Department of Botany and Plant Pathology, Oregon State University, Corvallis, Oregon, United States of America; CNRS/Université Joseph-Fourier, France

## Abstract

Ecologists have historically used species-area relationships (SARs) as a tool to understand the spatial distribution of species. Recent work has extended SARs to focus on individual-level distributions to generate individual species area relationships (ISARs). The ISAR approach quantifies whether individuals of a species tend have more or less species richness surrounding them than expected by chance. By identifying richness ‘accumulators’ and ‘repellers’, respectively, the ISAR approach has been used to infer the relative importance of abiotic and biotic interactions and neutrality. A clear limitation of the SAR and ISAR approaches is that all species are treated as evolutionarily independent and that a large amount of work has now shown that local tree neighborhoods exhibit non-random phylogenetic structure given the species richness. Here, we use nine tropical and temperate forest dynamics plots to ask: (*i*) do ISARs change predictably across latitude?; (*ii*) is the phylogenetic diversity in the neighborhood of species accumulators and repellers higher or lower than that expected given the observed species richness?; and (*iii*) do species accumulators, repellers distributed non-randomly on the community phylogenetic tree? The results indicate no clear trend in ISARs from the temperate zone to the tropics and that the phylogenetic diversity surrounding the individuals of species is generally only non-random on very local scales. Interestingly the distribution of species accumulators and repellers was non-random on the community phylogenies suggesting the presence of phylogenetic signal in the ISAR across latitude.

## Introduction

Understanding the determinants of species distributions and co-occurrence in hyper-diverse communities is a fundamental goal in ecology [1], [2]. Over the past several decades, mechanisms ranging from entirely deterministic to stochastic have been proposed to explain how large numbers of species are able to co-occur in complex and species-rich communities [3]. Traditional niche-based theory is often proposed as the predominant mechanism underlying the co-occurrence of plant species [4], [5]. However, it has been difficult to extend niche theory to species-rich communities.

In contrast to niche theory, the unified neutral theory [6] assumes that species abundances may drift neutrally over long periods of time and species-specific differences need not be invoked to explain co-occurrence [7], [8]. Both niche and neutral mechanisms are expected to leave particular spatial signatures in the distribution of biodiversity that can be detected by analyzing individual tree locations [9]. In particular, evidence indicating whether or not species have non-random spatial distributions can provide insights into the relative importance of niche or neutral processes as drivers of species coexistence in forests.

Recently, Wiegand et al. [10] proposed the framework that expands the concept of species-area relationships (SARs) to individual species-area relationships (ISARs) as means to determine whether individuals within a species are non-randomly distributed with respect to the distribution of other species. Specifically, the approach quantifies the species richness in concentric circles surrounding each individual of a species in a forest census plot, and assess whether the observed species richness is significantly higher, lower or no different from expected by spatial null models. Species with a higher and lower neighboring species richness than expected are referred to as a species accumulators and repellers, whereas species that are indistinguishable from the null expectation are referred to as neutral. Wiegand et al. [10] proposed that species accumulators tend to have positive biotic interactions within a community and species repellers tend to have negative biotic interactions. The positive and negative biotic interactions within a community may be derived from facilitation and competition respectively within abiotically homogeneous areas. Both accumulators and repellers have been taken as evidence for non-neutral or niche-based processes influencing the distribution and diversity of tree species in forest communities. Conversely if the species diversity around a target individual does not deviate significantly from that expected, this is taken as evidence for neutrality [10].

Despite the clear importance of the approach taken by Wiegand et al. [10] it is limited in that it treats all species as ecologically identical and evolutionarily independent. Species are obviously not ecologically identical and species interactions are governed by species function that is the result of evolution. Thus analyzing species co-occurrence using information about species beyond their binomials is likely to prove more powerful [11], [12], [13]. The importance of analyzing alternative dimensions of biodiversity such as phylogenetic diversity has been highlighted by a large number of recent studies that have reported the non-random phylogenetic structure of tree communities [14], [15], [16], [17], [18], [19], [20]. Thus a key goal in community ecology has been to extend research such as the ISAR approach of Wiegand et al. [10] to include other dimensions of biodiversity such as phylogenetic diversity.

To our knowledge, there has been no study investigating whether individual tree species in forest communities tend to be accumulators or repellers of phylogenetic diversity or whether species accumulators and repellers have non-random distributions on the phylogenetic tree itself. A key goal of the present study is to address whether individual species in forested communities around the world tend to be accumulators or repellers of phylogenetic diversity while controlling for their observed levels of neighboring species richness. To accomplish this we propose a framework for utilizing individual species and phylogenetic information to study tree-tree interactions across scales within forests. We introduce an individual phylogenetic area relationship (IPAR) that is analogous to the ISAR approach [10]. The IPAR quantifies the phylogenetic diversity (PD) of species within circular areas around a target individual of a species. The IPAR is then compared to that relationship expected at random given the observed species diversity in the neighborhood.

Here we apply the ISAR approach to a nine temperate and tropical forest dynamics plots (FDPs) to quantify whether there are any general ISAR patterns across tree communities that occur in vastly different environments and that range in species richness by an order of magnitude. We then extend the ISAR approach by incorporating phylogenetic information. In particular, we ask: (1) what is the phylogenetic diversity of the neighborhoods of accumulator, repeller and neutral species and is it any different from a random expectation?; and (2) what is the distribution of accumulator, repeller and neutral species on the phylogenetic tree? The results are to be discussed with respect to the co-occurrence of species, but more generally with respect to the spatial distribution of multiple axes of biodiversity in temperate and tropical tree assemblages.

## Methods

### Study Sites

The present study utilizes nine FDPs from temperate and tropical regions around the world (Table S1). Inside of each FDP all freestanding woody stems that have a diameter at breast height (d.b.h.) ≥1 cm are mapped, measured and identified [21], [22]. One of the FDPs (Wabikon Lake) is located in a temperate forest within the U.S.A. and the remainings are located in subtropical or tropical regions in Latin America, Asia and Africa. Four of the FDPs (Edoro-1, Edoro-2, Lenda-1, and Lenda-2) are located within a small region of the Democratic Republic of Congo. These four plots are not spatially contiguous and could not be spatially lumped together for our analyses. Interestingly the Lenda-1 and Lenda-2 FDPs are considered to be nearly monodominant stands of mbau (*Gilbertiodendron dewevrei* [Fabaceae]) with greater than 50% of the above ground biomass represented by this one species. The nearby Edoro-1 and Edoro-2 FDPs contain the mbau tree, but are not dominated by this species making the comparison of the Lenda and Edoro FDPs generally interesting. No specific permits were required for the described field studies, as no endangered or protected species was involved, and localities involved are not protected in any way.

### Individual Species-Area Relationships

The individual species-area relationship for a species can be defined as the expected number of species within nested circular areas around an average individual of that species. In other words, it describes the species richness around an average individual of a species at several neighborhood scales. The present study utilized nested circular neighborhoods with spatial scales ranging from 1 m - 50 m in radius in increments of 1 m. Therefore, in order to avoid edge effects we only sampled individuals of species that were at least 50 m from the nearest edge of the FDP.

The above ISAR approach reports the species richness within a neighborhood of an average individual at a particular spatial scale. For example, species X could on average have 10 species in its 15 m neighborhood when considering all individuals. Determining whether this average species richness is non-random requires a null model. We implemented the heterogeneous Poisson null model as described in Wiegand et al. [10]. Homogenous Poisson null models on the other hand randomly place all individuals of a species within the FDP that may be confounded by “first-order effects” where habitat associations of a species influencing its probability of occurrence are not considered. The heterogeneous Poisson model attempts to account for these first-order effects by constraining the random placement of individuals of a species by their known spatial distribution at broader scales thereby allowing for more robust inferences regarding positive and negative biotic interactions within abiotically homogeneous areas. In this study, as in previous work [10], we constrained the heterogeneous Poisson null model using an Epanechnikov kernal with a 50 m bandwidth. This process removes all spatial structure at scales less than 50 m during each iteration of the randomization, but preserves spatial structure at scales greater than 50 m. Using 999 iterations of the heterogenous Poisson null model we generated a null distribution of ISARs for each target species in the FDPs. The heterogeneous Poisson process is applied only on the target species while the distribution of the other species is kept intact when producing the null distribution of ISARs. The observed ISAR was compared to this null distribution. This determined whether the average individual of a species with a given spatial neighborhood scale has more or fewer species in its neighborhood than expected given the null model. Those species with significantly more than expected species at a spatial scale were designated ‘accumulator species’. Those species with significantly fewer than expected species at a spatial scale were designated ‘repeller species’. Finally those that had neighborhood species richness values indiscernible from the random expectation were designated ‘neutral species’. It is important to note that a species is designated as an accumulator, repeller or neutral at each of the 50 spatial scales utilized. Thus, a species could have any one of these designations at a given scale. The analyses were repeated using two different approaches. One where all species that have individuals ≥1 cm dbh were utilized and the other using all species that had ≥70 individuals ≥1 cm dbh.

### Phylogenetic Tree Reconstruction

A phylogenetic tree was constructed for each FDP used in this study. The phylogenetic trees were constructed using the online informatics tool Phylomatic [23]. Phylomatic constructs a phylogeny using the Angiosperm Phylogeny Group III family-level tree [24] and data based phylogenetic relationships among genera and species. This phylogenetic backbone next has species relationships estimated using their taxonomy resulting in a phylogenetic tree with little resolution among con-generic species and often among con-familial genera. We calibrated the phylogenetic branch lengths to time using the ‘bladj’ algorithm in the software Phylocom [25], which provides simplistic estimates of nodal dates. Despite the weaknesses of generating such a phylogeny it is suitable for our current work for the following reasons. First, as described below, our analyses use the PD statistic to measure phylogenetic diversity of individual neighborhoods. Recent work by Swenson [26] has shown that a lack of terminal resolution in phylogenies such as those generated by Phylomatic does not introduce any large biases into the PD metric. Second, the crudely estimated branch lengths generated by the ‘bladj’ algorithm are substantially more informative than setting all branch lengths to one particularly when the phylogenetic composition of the assemblages spans nearly the entire angiosperm phylogeny in most forests. Ideally future work will incorporate molecular phylogenies, which are increasingly becoming feasible for diverse tree assemblages [18], [27].

### Individual Phylogenetic-Area Relationships

The ISAR approach described above allows one to designate a species as an accumulator, repeller or neutral species at each spatial scale. The composition of those species in the neighborhoods, though, is not quantified under the ISAR approach. For example, if a species is known to accumulate other species into its neighborhood, we do not know whether or not those accumulated species are phylogenetically similar or dissimilar from one another. In order to answer this question we quantified the phylogenetic diversity (PD) [28] of an average individual of a species using the neighborhood spatial scales of 1–10 m, 15 m, 20 m, 30 m, 40 m and 50 m. Since PD is related to the species richness, we wanted to know whether the observed PD is any higher or lower than that expected given the observed species richness. We utilized a phylogeny defined for each forest and shuffled the names of species across the tips of the phylogeny 999 times as null model. In other words a single phylogeny was made for each forest and the null model was generated from that phylogeny. Therefore species from other forests in this study were not included in the null models. This resulted in a null distribution of the PD of an average individual of a species. We compared the observed PD of an average individual of a species to this null distribution and calculated a standardized effect size (S.E.S. PD) by subtracting the observed value from the mean of null distribution and dividing that value by the standard deviation of the null distribution. S.E.S. PD was calculated as following:



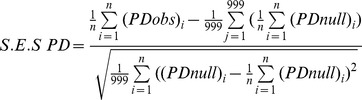
.*i* is the number of individuals for target species. *j* is the number of null values. PD_obs_ is the observed value of phylogenetic diversity. The PD_null_ are the simulated values of phylogenetic diversity. Thus positive S.E.S PD values indicated that the PD of the average individual of a species was higher than expected, while negative S.E.S. PD values indicated a lower than expected PD around the average individual. This approach allowed us to differentiate between, for example, accumulator species that accumulate phylogenetically diverse neighborhoods or phylogenetically poor neighborhoods. The null model utilized for this analysis is not a spatial null model such as the null model used in the ISAR analyses. This was done because we were primarily interested in the observed species composition around an average individual of a species and whether it was phylogenetically non-random. A spatial null model like that used for ISAR analyses does not permit a direct answer to this question and would not all us to disentangle the ISAR results from the S.E.S. PD results.

### Phylogenetic Distribution of Accumulator, Repeller and Neutral Species

A goal of this study was to quantify the phylogenetic distribution of accumulator, repeller and neutral species. For example, we ask whether accumulator species tend to be closely related to one another, distantly related to one another or have patterns of relatedness that are no different from a random expectation. Answering this question is akin to asking what the phylogenetic dispersion of accumulators is given a phylogenetic tree. To measure the phylogenetic dispersion of accumulators, repellers or neutral species we also used the two most widely used standardized effect size metrics of phylogenetic dispersion – the Net Relatedness Index (NRI) and the Nearest Taxon Index (NTI) [14]. The NRI measures the basal phylogenetic dispersion of an assemblage (e.g. accumulator species in a FDP) by comparing the observed mean pairwise phylogenetic distance between species in an assemblage to the random expectation given a null model. The NTI measures the terminal phylogenetic dispersion of an assemblage by comparing the observed mean nearest phylogenetic neighbor distance between species in an assemblage to the random expectation given a null model. The null model implemented was to shuffle the names of species on the phylogenetic tree 999 times and to recalculate the pairwise and nearest neighbor metrics each time to create a null distribution. Negative values of NRI or NTI indicated that the assemblage (e.g. accumulators) was overdispersed on the FDP phylogeny while positive values of NRI or NTI indicated that the assemblage was clustered on the phylogeny. In other words negative values indicated species were not closely related whereas positive values indicated species were closely related. We calculated the NRI and NTI for the accumulator, repeller and neutral species assemblages in each FDP at each of the 50 spatial scales used in the ISAR calculations. The analysis was performed using R package ‘Picante’ [29], [30].

We used the parsimony Sankoff score [31] to test for phylogenetic signal in the status of species accumulator, repeller and neutral species. To test for significant phylogenetic signal, we randomly arrayed the species status on the community phylogeny 999 to generate a null distribution from which a *p* value could be calculated. The analysis was performed using R package ‘Phangorn’ [32]. Fisher’s exact test was used to examine the correlation between the status of species accumulator, repeller and neutral species and the status of phylogenetic accumulator, repeller and neutral species, where two-tailed *P* values of <0.05 were determined.

We also used the D statistic developed by Fritz and Purvis [33] to measure the phylogenetic signal in a binary trait. Specifically, we scored species as either an accumulator or a repeller at each spatial scale. The D value is 1 if the distribution of the binary trait is random with respect to phylogeny and greater than 1 if the distribution of the trait is more labile than the random expectation. The D value is 0 if the binary trait is distributed as expected under the Brownian motion model of evolution and less than 0 if the binary trait is less variable than the Brownian motion expectation [33]. We performed 1000 permutations of the binary trait on the phylogeny to determine the significance of the observed D value on each of the spatial scales from 1–50 m in each plot. The details for how to calculate D and assess its significance can be seen in Fritz and Purvis [33]. This analysis was performed using R package ‘Caper’ [34].

## Results

### Individual Species-Area Relationships

**Figure 1:**
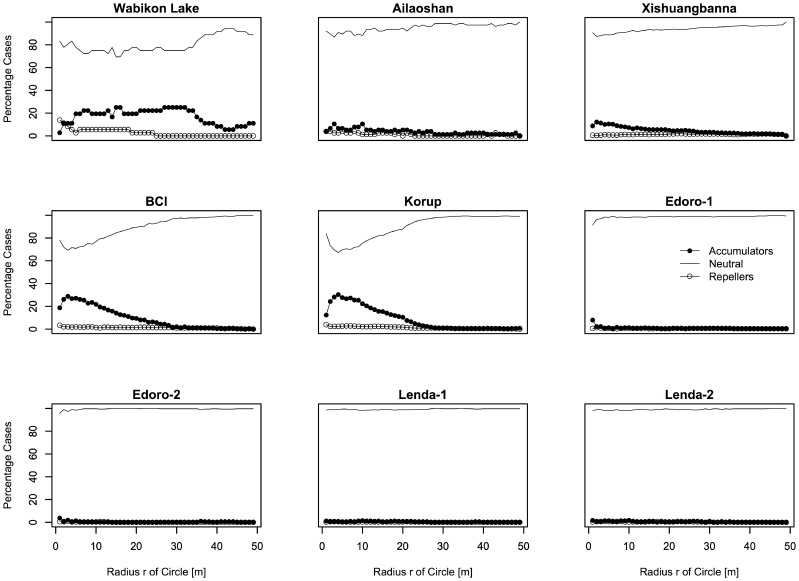
Proportion of significant species diversity accumulators, repellers and neutral species for the species with individuals ≥1 cm dbh in the nine plots.

**Figure 2:**
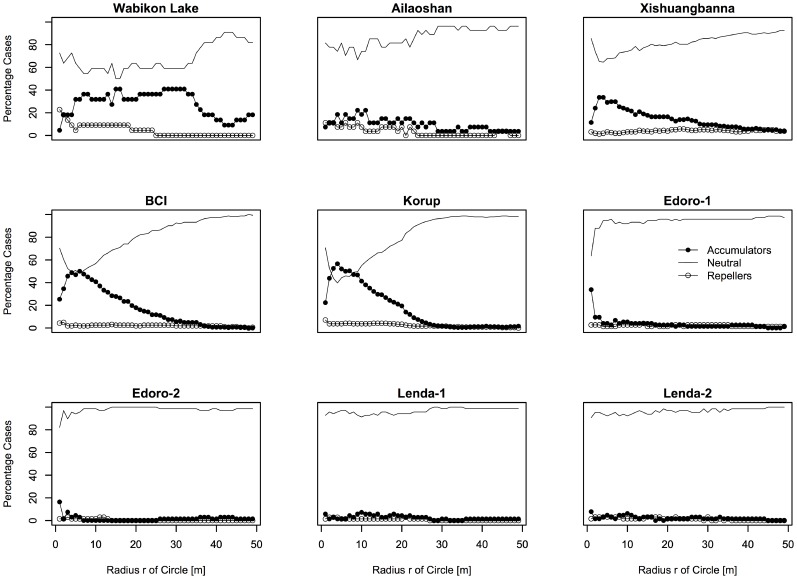
Proportion of species diversity accumulators, repellers and neutral species for the species having ≥70 individuals ≥1 cm in the nine plots.

The proportion of species diversity accumulator, repeller and neutral species at multiple spatial scales varied greatly across tropical and temperate tree communities (Figures 1 and 2). The proportion of species diversity accumulators or repellers was remarkably similar among four plots of Congo. There were nearly no species diversity accumulators and repellers found at scales *r* from 1–50 m, the most species have neutral effects on neighbor species. Other tropical plots except Congo generally have over 10 percent of their species being accumulators at scale of <20 m. Subtropical plot generally have less than 10 percent of the species being accumulators and repellers at scale *r*<50 m. Species in the temperate plot have a contrary pattern. Specifically, the Wabikon Lake plot had more diversity accumulators at large neighborhood scales than other plots (Figures 1 and 2).

### Individual Phylogenetic-Area Relationships

**Figure 3:**
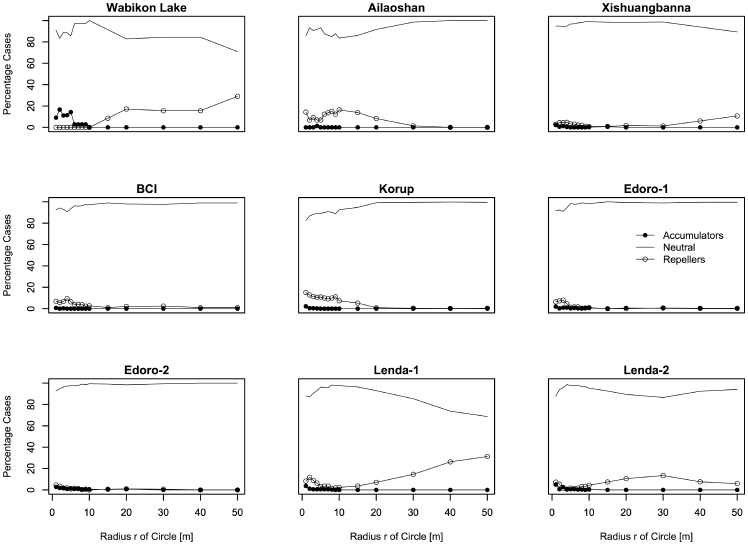
Proportion of phylogenetic diversity accumulators, repellers and neutral species for the species with individuals ≥1 cm dbh in the nine plots.

**Figure 4:**
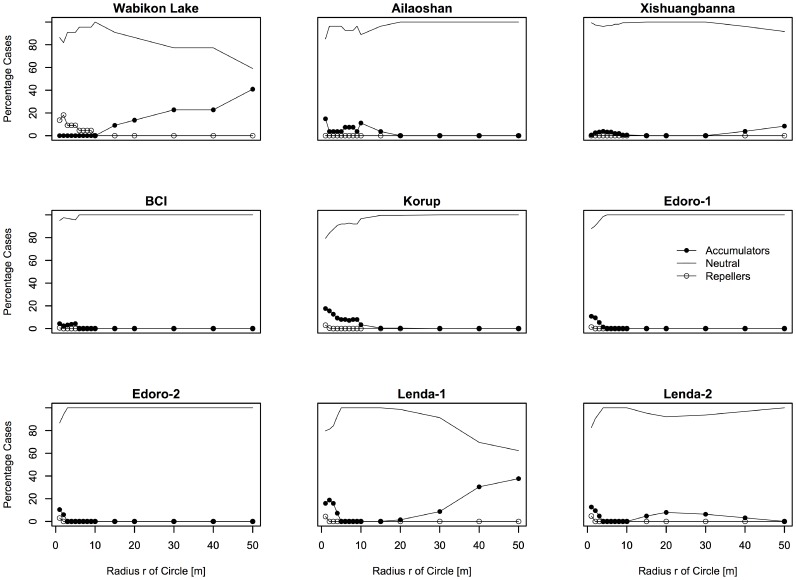
Proportion of phylogenetic diversity accumulators, repellers and neutral species for the species having ≥70 individuals ≥1 cm in the nine plots.

The proportion of phylogenetic diversity accumulator, repeller and neutral species were similar in all plots at similar scales irrespective of whether we analyzed all species or only the most common species (Figures 3 and 4). The majority of the IPARs were neutral for species across the spatial scales investigated (Figures 3 and 4). There were only few phylogenetic diversity repellers identified on fine spatial scales (*r*<5 m), whereas most species were neutral on meso-scales. Interestingly, four plots (Wabikon Lake, Xishuangbanna, Lenda-1 and Lenda-2) had more than 10 percent of their species identified as phylogenetic diversity repellers at larger spatial scales (scales over 20 m).

### Phylogenetic Dispersion of Accumulator, Repeller and Neutral Species

In general, the status of species accumulator, repeller and neutral species and the status of phylogenetic accumulator, repeller and neutral species are independent from each other (Table S2). There was significant phylogenetic signal in the status of species accumulator, repeller and neutral species on local scales from 1–50 m across the nine plots (Table S3). Specifically, based on the results of NRI and NTI, species diversity accumulators were phylogenetically clustered in the nine plots on the finest spatial scale (Table S4). Species repellers were phylogenetically clustered on the scales less than 20 m in Korup and Xishuangbanna plots. However, there was in general no consistent trend of phylogenetic distribution of species repellers across the nine plots (Table S5).

**Figure 5:**
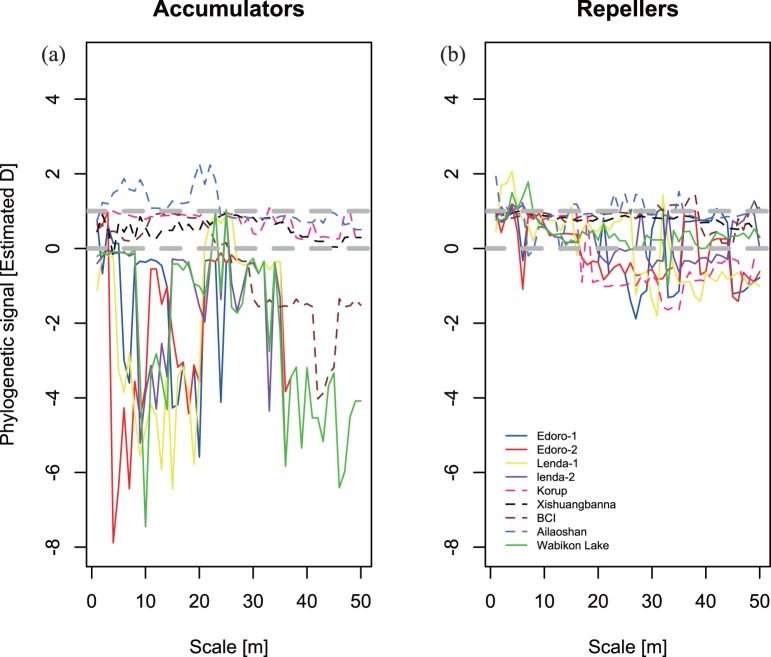
The phylogenetic signal of species diversity accumulators and repellers on each scale from 0–50 m in the nine forest dynamics plots, using D statistic developed by Fritz and Purvis (2010). Grey horizontal lines represent random expectation (D = 1) and Brownian expectation (D = 0).

Using the D statistic for phylogenetic signal in a binary trait, the dispersion of species accumulators had phylogenetic signal in four plots in Congo (Edoro-1, Edoro-2, Lenda-1 and Lenda-2) and the Wabikon Lake plot on local scales from 1–50 m (Figure 5, D<0). The dispersion of species accumulators had no phylogenetic signal on the scales<30 m in Ailao plot (Figure 5a, D>1), and had phylogenetic signal on the scales>30 m in BCI plot (Figure 5a, D<0). The phylogenetic signal in species accumulators in Xishuangbanna and Korup plots was weak on scales from 1–50 m (Figure 5a, 0< D<1). There was in general no consistent trend in the phylogenetic distribution of species repellers across the nine plots (Figure 5b). In general, the observed phylogenetic patterns differed significantly from the random and Brownian Motion expectations (*p*<0.05).

## Discussion

In this study, we first quantified the individual species-area relationship (ISAR) in nine forest plots across latitude and then put this result into a phylogenetic context. This integration was first achieved by quantifying whether the neighborhoods of species were more or less phylogenetically diverse than expected using a null modeling approach. This generated what we term individual phylogenetic-area relationships (IPARs). The second way we integrated the ISAR and phylogenies was to quantify whether species accumulators and repellers had non-random distributions on community phylogenies. In the following we discuss the results of our analyses.

### Scale Dependence of Biological Mechanisms Underlying ISAR and IPAR

The present study uncovered strong spatial scale dependence across latitude in the degree to which species behave non-randomly or randomly. In particular, species were significant accumulators or repellers of diversity only on local spatial scales (<20 m) and generally behaved neutrally on larger spatial scales (Figure 1). This result also held when only considering common species (species with ≥70 individuals) (Figure 2). This result is important because it confirms the importance of scale dependence in co-occurrence patterns in tree communities where the importance of species interactions generally cannot be detected at spatial scales larger than 20 m in distance [16], [17], [19]. Further it strengthens the inferences of Wiegand et al. [10] who only analyzed two forest plots and found that species-specific effects on local diversity were limited to generally within 20 m from the focal individual. Ultimately, these findings demonstrate the importance of non-neutral processes governing co-occurrence on fine scales across enormous species richness and climatic gradients and that these processes leave a strong signature on the spatial distribution of individuals in tree communities.

The IPAR results also uncovered strong spatial scale-dependence across latitude. In particular, species were designated to be phylogenetic accumulators only on local spatial scales (<20 m) and generally behaved neutrally on larger spatial scales except in the Wabikon plot (Figures 3 and 4). Even when using the individuals ≥10 cm, there are similar trend across latitude aside from the Wabikon plot (Figures S3 and S4). This result is consistent with previous work that stresses the importance of non-random processes on the phylogenetic structure of tree communities on local scales [17], [19]. Phylogenetic diversity accumulators are hypothesized to be evidence of facilitation among distantly related species, while phylogenetic diversity repellers are hypothesized to be target species where closely related species filter into the same neighboring environment. Whether or not these results are generated by abiotic or biotic interactions is difficult to infer given our lack of knowledge regarding niche evolution (i.e. conserved or convergent) in these plots, but we can safely infer that non-neutral niche-based processes are likely to be generating the observed patterns on fine scales. Conversely, on larger spatial scales our IPAR results were no different from a random expectation. These results could be generated by one of three mechanisms. First, neutral processes may dominate at these larger spatial scales within forest plots. Second, non-random abiotic and biotic processes may be operating simultaneously to produce an apparently random pattern [35]. Third, analyses at these larger spatial scales may lack the statistical power necessary to reject a null expectation [19].

### Latitude Gradient of Biological Mechanisms Underlying ISAR

We further investigated the ISAR results with respect to whether the forest plot was tropical, subtropical or temperate. We found there was a trend in ISAR results across latitude. In general, tropical tree communities tended to have more species accumulators than subtropical and temperate tree communities and the subtropical and temperate plots had more repellers when we analyzed the target species with all individuals ≥1 cm dbh (Figure 1). This result was in some ways surprising as the general inference from such results is that there are more negative interactions in the temperate zone and subtropics than in the tropics, which is contrary to many hypotheses regarding the latitudinal gradient in species richness.

### The Biological Mechanism Underlying the Phylogenetic Signal in ISAR

Our final set of analyses asked whether species richness accumulators and repellers were non-randomly distributed on the phylogeny. Based on the D statistic, we found that the ‘trait’ of species accumulation has phylogenetic signal on local scales and we also found species richness accumulators were generally clustered on the community phylogeny (Figure 5a). Thus species accumulators are generally closely related. In contrast, the ‘trait’ of species repelling was found to have relatively weak phylogenetic signal on local scales and species richness repellers had weak clustering on the community phylogeny (Figure 5b). Thus, we may expect that lineages in species poor communities will be on average distantly related and species rich communities will be composed of species that are both distantly and closely related.

In sum, these results demonstrate the past evolutionary history in part explains why some species tend to be accumulators of species richness in temperate and tropical tree communities. We also provided detailed lineage-specific information regarding species accumulators and repellers. Specifically, most species accumulators came from the Rosid clade, and species repellers were overdispersed in core eudicots (Table S6) [24]. A major hurdle in inferring the co-existence and assembly mechanisms from these lineages is the lack of species functional information. However, the frequent clustering of species accumulators on the phylogeny across all nine plots may be driven by facilitation.

### Methodological Issues

Under classic assembly theory along abiotic gradients we do not expect negative and positive interactions to have the same degree of importance at particular points on the gradient. Considering the “first-order effects” where habitat association increases or decreases the likelihood that an individual will occur at a given location, we applied heterogeneous Poisson null model in which the individuals of the target species are distributed in accordance with spatially variable intensity to control the effect of habitats filtering. For spatially explicit data, like tree distribution data in the nine plots used in this study, a heterogeneous Poisson process has some potential to be a null model factoring out the effect of habitat heterogeneity.

Although a heterogeneous Poisson null model has never been evaluated against simulated datasets to assess whether it is effective, it has been successfully applied in spatial point pattern analyses regarding species associations [10], [36], [37]. For example, Wiegand et al. [10] estimated the intensity function by using an Epanechnikov kernel with a bandwidth of 50 m, which removes all potential spatial structure in the pattern of the target species at scales<50 m, but maintains the spatial structure at scales>50 m. In other words, a heterogeneous Poisson null model with a bandwidth of 50 m can in theory factor out the impact of habitat filtering finer than 50 m. Following Wiegand et al. [10], we calculated all scale-dependent functions with steps of 1 m and up to a maximal scale of 50 m. If we continue to reduce the heterogeneous Poisson bandwidth, the process of randomizing the locations of the trees of the target species will becomes a homogeneous Poisson. The outcome of analyses with the homogeneous Poisson null model may be confounded by “first-order effects” where habitat association increases or decreases the likelihood that an individual will occur at a given location.

To date research into ISARs has tried to remove the importance of abiotic filtering beyond trying to control for its effect in null models, but these null models generally only consider abiotic filtering on spatial scales 50 m and greater [10]. While this null modeling approach likely does remove some of the abiotic filtering signature in tree assemblages, field research has shown that abiotic filtering of traits and lineages occurs on scales finer than 50 m [19], [20], [35] in these forests suggesting that abiotic filtering could leave a pattern of many species repellers.

We also recognized the effects of spatial aggregation and high local density caused by limited dispersal on designating a species to be species accumulator or repeller. If a species typically occurs spatially aggregated at a high local density, much of the living space surrounding its individuals will be occupied by conspecifics, leaving less space for heterospecific species. In this case, target species might be more easily detected as repellers when we use individuals ≥1 cm dbh in our analyses, because this size class includes almost all saplings which tend to be spatially aggregated at a high local density caused by limited dispersal. Without controlling for the effect of limited dispersal, we might observe a higher proportion of repellers than that controlling the effect of limited dispersal. In order to control the effects of limited dispersal, we re-analyzed the target species only using the individuals ≥10 cm dbh (Figures S1 and S2), which may be relatively spatially non-aggregated and less possible with high local density as shown in Xishuangbanna plot [38]. However, we observed a higher proportion of species repellers on small scales (<20 m) in tropical forests when only using the individuals ≥10 cm dbh (Figures S1 and S2) than that using all individuals ≥1 cm dbh (Figures 1 and 2). If dispersal limitation did exert an effect on designating a species to be species accumulator or repeller, there should be higher proportion of species repellers when using all individuals ≥1 cm dbh, but this was not confirmed when we compared the results between the individuals in a larger size class and all individuals. In the temperate forests, however, there are higher proportions of species repellers on small scales (<10 m) when using all individuals ≥1 cm dbh (Figures 1 and 2) than ≥10 cm dbh (Figures S1 and S2), which indicated that limited dispersal might increase the probability of a target species to be detected as a species repeller. In sum, the results in our analyses when using all individuals ≥1 cm dbh might be a balance between larger size classes and smaller size classes which might show different interactions with other species around them and thus lead to different designations of the target species in different size classes. Therefore, we suggest that all individuals of a target species should be divided into different size classes to analyze their interaction with other species around them in the same size class and future null model based work should attempt to fix the observed dispersal limitation or approximates it more closely.

A weakness of the ISAR approach is that it treats all species as identical. Indeed an individual could have a neighborhood of 10 closely related species or 10 distantly related species and the ISAR would treat both scenarios as the same. This lack of information may limit the inferences that can be drawn from ISARs. This seems particularly likely in tree communities where researchers have found that the phylogenetic structure of assemblages is often non-randomly more or less diverse than that expected when controlling for species richness levels [14], [16], [17]. Here we have proposed an individual phylogenetic-area relationship (IPAR), which asks whether the phylogenetic diversity (PD) in the circle around individuals of a species is higher or lower than that expected given the observed species richness.

In this study, IPAR is tested using a null model shuffling species in the phylogeny to detect if PD is higher or lower than expected after randomization. Hardy [39] has suggested the potential importance of the distribution of species abundances in the phylogeny when constructing community phylogenetic null models. Specifically, phylogenetic signal in abundances may greatly decrease the effectiveness of null models that shuffle names on the phylogeny. Recent work by Mi et al. [40] has found that the Blomberg’s *K* statistic for each of 15 temperate and tropical FDPs is less than 1 for the trait of abundance, indicating abundance is weakly conservative on community phylogeny. In other words, abundance is more variable among species than expected given their shared branch lengths and a random walk of trait evolution. Thus, there should not have significant negative effects of species abundance on shuffling the species in the phylogeny.

### Conclusions

Our results suggest that biotic interactions on individual-level distributions in communities are strongest at spatial scales r<30 m in the nine tropical and temperate forests. This lends support to the idea that non-neutral processes such as competition and facilitation may leave a detectable signature at small-scales spatial pattern of species diversity but result in stochastic patterns at larger-scales. This study also highlights how analyzing alternative dimensions of biodiversity, such as phylogenetic diversity, may help us understand the co-occurrence of species in diverse assemblages. In particular, we have shown that the phylogenetic distribution of species accumulators and repellers in forests is strongly non-random indicating the importance of past evolutionary history in dictating the ecological interactions we presently observe.

## Supporting Information

Figure S1 **Proportion of species diversity accumulators, repellers and neutral species for the species with individuals** ≥**10 cm dbh in the nine plots.**
(EPS)

Figure S2 **Proportion of significant species diversity accumulators, repellers and neutral species for the species having ≥70 individuals** ≥**10 cm dbh in the nine plots.**
(EPS)

Figure S3 **Proportion of significant phylogenetic diversity accumulators, repellers and neutral species for the species with individuals** ≥**10 cm dbh in the nine plots.**
(EPS)

Figure S4 **Proportion of significant phylogenetic diversity accumulators, repellers and neutral species for the species having ≥70 individuals** ≥**10 cm dbh in the nine plots.**
(EPS)

Table S1 **The basic information of the nine forest dynamics plots utilized in this study.**
(DOC)

Table S2 **The** ***P*** **value of fisher’s exact test for the correlation between the status of species accumulator, repeller and neutral species and the status of phylogenetic accumulator, repeller and neutral species on local scales in the nine forest plots.**
(DOCX)

Table S3 **The test for phylogenetic signal in the status of species accumulator, repeller and neutral species on local scales from 1 m to 50 m using parsimony Sankoff score.**
(DOCX)

Table S4 **The phylogenetic dispersion of species diversity accumulator at each scale of the nine forest dynamics plots based on NRI and NTI.**
(DOCX)

Table S5 **The phylogenetic dispersion of species diversity repeller at each scale of the nine forest dynamics plots based on NRI and NTI.**
(DOCX)

Table S6 **The lineage information for the target species designated to be species accumulator or repeller on certain scales in the nine forest plots.** This table does not include the target species designated to be accumulator on some scales and repeller on some other scales.
(DOCX)

## References

[1] Ricklefs RE (1987) Community diversity: relative roles of local and regional processes. Science 235: 167–171. 17778629 10.1126/science.235.4785.167

[2] Chesson P (2000) General theory of competitive coexistence in spatially varying environments. Theor Popul Biol 58: 211–237. 11120650 10.1006/tpbi.2000.1486

[3] Wright JS (2002) Plant diversity in tropical forests: a review of mechanisms of species coexistence. Oecologia 130: 1–14. 28547014 10.1007/s004420100809

[4] Tilman D (1982) Resource competition and community structure. Monographs in population biology. Princeton: Princeton Univ Press. 7162524

[5] Silvertown J, Dodd ME, Gowing DJG, Mountford JO (1999) Hydrologically defined niches reveal a basis for species richness in plant communities. Nature 400: 61–63.

[6] Hubbell SP (2001) The unified neutral theory of biodiversity and biogeography. Princeton: Princeton Univ Press. 10.1016/j.tree.2011.03.024 21561679

[7] Hubbell SP (2005) Neutral theory in community ecology and the hypothesis of functional equivalence. Funct Ecol 19: 166–172.

[8] Hubbell SP (2006) Neutral theory and the evolution of ecological equivalence. Ecology 87: 1387–1398. 16869413 10.1890/0012-9658(2006)87[1387:ntateo]2.0.co;2

[9] Hubbell SP, Ahumada JA, Condit R, Foster RB (2001) Local neighborhood effects on long-term survival of individual trees in a neotropical forest. Ecol Res 16: 859–875.

[10] Wiegand T, Gunatilleke CVS, Gunatilleke IAUN, Huth A (2007) How individual species structure diversity in tropical forests. Proc Natl Acad Sci USA 104: 19029–19033. 18024595 10.1073/pnas.0705621104 PMC2141902

[11] Webb CO, Ackerly DD, McPeek MA, Donoghue MJ (2002) Phylogenies and community ecology. Annu Rev Ecol Syst 33: 475–505.

[12] McGill BJ, Enquist BJ, Weiher E, Westoby M (2006) Rebuilding community ecology from functional traits. Trends Ecol Evol 21: 178–185. 16701083 10.1016/j.tree.2006.02.002

[13] Swenson NG (2011) The role of evolutionary processes in producing biodiversity patterns, and the interrelationships between taxonomic, functional and phylogenetic biodiversity. Am J Bot 98: 472–480. 21613140 10.3732/ajb.1000289

[14] Webb CO (2000) Exploring the phylogenetic structure of ecological communities: an example for rain forest trees. Am Nat 156: 145–155. 10856198 10.1086/303378

[15] Cavender-Bares J, Keen A, Miles B (2006) Phylogenetic structure of Floridian plant communities depends on taxonomic and spatial scale. Ecology 87: 109–122. 10.1890/0012-9658(2006)87[109:psofpc]2.0.co;2 16922307

[16] Swenson NG, Enquist BJ, Pither J, Thompson J, Zimmerman JK (2006) The problem and promise of scale dependency in community phylogenetics. Ecology 87: 2418–2424. 17089650 10.1890/0012-9658(2006)87[2418:tpapos]2.0.co;2

[17] Swenson NG, Enquist BJ, Thompson J, Zimmerman JK (2007) The influence of spatial and size scale on phylogenetic relatedness in tropical forest communities. Ecology 88: 1770–1780. 17645023 10.1890/06-1499.1

[18] Kress WJ, Erickson DL, Jones FA, Swenson NG, Perez R, et al (2009) Plant DNA barcodes and a community phylogeny of a tropical forest dynamics plot in Panama. Proc Natl Acad Sci USA 106: 18621–18626. 19841276 10.1073/pnas.0909820106 PMC2763884

[19] Kraft NJB, Ackerly DD (2010) Functional trait and phylogenetic tests of community assembly across spatial scales in an Amazonian forest. Ecol Monogr 80: 401–422.

[20] Swenson NG, Erickson DL, Mi XC, Bourg NA, Forero-Montaña J, et al (2012) Phylogenetic and functional alpha and beta diversity in temperate and tropical tree communities. Ecology 93: S112–S125. 10.1890/11-1180.1 22624204

[21] Condit R (1998) Tropical forest census plots. Berlin: Springer.

[22] Losos EC, Leigh EG (2004) Tropical forest diversity and dynamics: findings from a network of large-scale tropical forest plots. Chicago: Chicago Univ Press.

[23] Webb CO, Donoghue MJ (2005) Phylomatic: tree assembly for applied phylogenetics. Molec Ecol 5: 181–183.

[24] The angiosperm phylogeny group (2009) An update of the Angiosperm Phylogeny Group classification for the orders and families of flowering plants: APG III. Bot J Linn Soc 161: 105–121.

[25] Webb CO, Ackerly DD, Kembel SW (2008) Phylocom: software for the analysis of phylogenetic community structure and trait evolution. Bioinformatics 24: 2098–2100. 18678590 10.1093/bioinformatics/btn358

[26] Swenson NG (2009) Phylogenetic resolution and quantifying the phylogenetic diversity and dispersion of communities. PLoS ONE 4: e4390. 19194509 10.1371/journal.pone.0004390 PMC2633039

[27] Kress WJ, Erickson DL, Swenson NG, Thompson J, Uriarte M, et al (2010) Advances in the use of DNA barcodes to build a community phylogeny for tropical trees in a Puerto Rican forest dynamics plot. PLoS ONE 5: e15409. 21085700 10.1371/journal.pone.0015409 PMC2976767

[28] Faith DP (1992) Conservation evaluation and phylogenetic diversity. Biol Conserv 61: 1–10.

[29] R Development Core Team (2012) R: A language and environment for statistical computing. R Foundation for Statistical Computing. Available: http://www.R-project.org. Accessed 2012 Aug 9.

[30] Kembel SW, Ackerly DD, Blomberg SP, Cornwell WK, Cowan PD (2010) Picante: R tools for integrating phylogenies and ecology. Bioinformatics 26: 1463–1464. 20395285 10.1093/bioinformatics/btq166

[31] Maddison WP, Slatkin M (1991) Null models for the number of evolutionary steps in a character on a phylogenetic tree. Evolution 45: 1184–1197. 28564173 10.1111/j.1558-5646.1991.tb04385.x

[32] Schliep KP (2011) Phangorn: phylogenetic analysis in R. Bioinformatics. 27: 592–593. 10.1093/bioinformatics/btq706 PMC3035803 21169378

[33] Fritz SA, Purvis A (2010) Selectivity in mammalian extinction risk and threat types: a new measure of phylogenetic signal strength in binary traits. Conserv Biol 24: 1042–1051. 20184650 10.1111/j.1523-1739.2010.01455.x

[34] Orme D, Freckleton R, Thomas G, Petzoldt T, Fritz S et al. (2012) caper: comparative analyses of phylogenetics and evolution in R. R package version 0.5. R Foundation for Statistical Computing. Available: http://CRAN.R-project.org/package=caper. Accessed 2012 Dec 9.

[35] Swenson NG, Enquist BJ (2009) Opposing assembly mechanisms in a Neotropical dry forest: implications for phylogenetic and functional community ecology. Ecology 90: 2161–2170. 19739378 10.1890/08-1025.1

[36] Wang XG, Wiegand T, Hao ZQ, Li BH, Ye J, et al (2010) Species associations in an old-growth temperate forest in north-eastern China. J Ecol 98: 674–686.

[37] Wiegand T, Huth A, Getzin S, Wang X, Hao Z, et al (2012) Testing the independent species’ arrangement assertion made by theories of stochastic geometry of biodiversity. Proc R Soc B 279: 3312–3320. 10.1098/rspb.2012.0376 PMC3385721 22593112

[38] Lan GY, Hu YH, Cao M, Zhu H (2011) Topography related spatial distribution of dominant tree species in a tropical seasonal rain forest in China. Forest Ecol Manag 262: 1507–1513.

[39] Hardy OJ (2008) Testing the spatial phylogenetic structure of local communities: statistical performances of different null models and test statistics on a locally neutral community. J Ecol 96: 914–926.

[40] Mi X, Swenson NG, Valencia R, Kress WJ, Erickson DL, et al (2012) The contribution of rare species to community phylogenetic diversity across a global network of forest plots. Am Nat 180: E17–E30. 22673660 10.1086/665999

