# Supplementary material for: A Phylogenetic Perspective on the Individual Species-Area Relationship in Temperate and Tropical Tree Communities
Source: PLoS One. 2013 May 1;8(5):e63192. doi: 10.1371/journal.pone.0063192 (PMC3641141; doi:10.1371/journal.pone.0063192)
Supplement: Table S1 — The basic information of the nine forest dynamics plots utilized in this study. (DOC) [file pone.0063192.s005.doc]

**Table S1.** The basic information of the nine forest dynamics plots utilized in this study.

| Forest  Dynamics Plot | Country | Forest Type | Latitude | Longitude | Area (ha) | Total Species Richness | Total Individual Trees |
| --- | --- | --- | --- | --- | --- | --- | --- |
| Ailaoshan | China | Subtropical Moist Forest | 24.32 N | 102.01E | 6 | 76 | 17829 |
| Barro Colorado Island (BCI) | Panama | Tropical Moist Forest | 9.154 N | -79.846E | 50 | 299 | 208387 |
| Edoro-1 | Democratic Republic of Congo | Tropical Rain Forest | 1.437 N | 28.5826E | 10 | 315 | 75019 |
| Edoro-2 | Democratic Republic of Congo | Tropical Rain Forest | 1.437 N | 28.5826E | 10 | 326 | 85123 |
| Korup | Cameroon | Tropical Rain Forest | 5.074 N | 8.854E | 50 | 494 | 327044 |
| Lenda-1 | Democratic Republic of Congo | Tropical Rain Forest | 1.437 N | 28.5826E | 10 | 349 | 68387 |
| Lenda-2 | Democratic Republic of Congo | Tropical Rain Forest | 1.437 N | 28.5826E | 10 | 300 | 64286 |
| Wabikon Lake | U.S.A. | Temperate Deciduous Forest | 45.551 N | -88.796E | 25 | 36 | 49585 |
| Xishuangbanna | China | Tropical Rain Forest | 21.612 N | 101.574E | 20 | 468 | 95611 |
