# Supplementary material for: A Phylogenetic Perspective on the Individual Species-Area Relationship in Temperate and Tropical Tree Communities
Source: PLoS One. 2013 May 1;8(5):e63192. doi: 10.1371/journal.pone.0063192 (PMC3641141; doi:10.1371/journal.pone.0063192)
Supplement: Table S2 — The P value of fisher’s exact test for the correlation between the status of species accumulator, repeller and neutral species and the status of phylogenetic accumulator, repeller and neutral species on local scales in the nine forest plots. (DOCX) [file pone.0063192.s006.docx]

**Table S2.** The *P* value of fisher’s exact test for the correlation between the status of species accumulator, repeller and neutral species and the status of phylogenetic accumulator, repeller and neutral species on local scales in the nine forest plots.

| Radius (m) | *P* Value | | | | | | | | |
| --- | --- | --- | --- | --- | --- | --- | --- | --- | --- |
|  | Ailaoshan | BCI | Edoro-1 | Edoro-2 | Korup | Lenda-1 | Lenda-2 | Wabikon Lake | Xishuangbanna |
| r=1 | 0.036 | 0.037 | 0.604 | 1 | 0.003 | 1 | 0.348 | 0.031 | 1 |
| r=2 | 0.033 | 0.049 | 1 | 1 | 0.048 | 1 | 1 | 0.246 | 0.032 |
| r=3 | 0.024 | 0.003 | 0.374 | 1 | 0.049 | 0.335 | 1 | 0.019 | 0.019 |
| r=4 | 0.035 | 0.041 | 1 | 1 | 0.054 | 1 | 1 | 0.005 | 0.007 |
| r=5 | 0.004 | 0.029 | 1 | 1 | 0.01 | 1 | 1 | 0.029 | 0.007 |
| r=6 | 0.064 | 0.012 | 1 | 1 | 0.008 | 1 | 1 | 0.027 | 0.004 |
| r=7 | 0.058 | 0.026 | 1 | 1 | 0.005 | 1 | 1 | 0.029 | 0.003 |
| r=8 | 0.026 | 0.015 | 1 | 1 | 0.002 | 1 | 1 | 0.11 | 0.062 |
| r=9 | 0.020 | 0.012 | 1 | 1 | 0.023 | 1 | 1 | 0.143 | 1 |
| r=10 | 0.050 | 0.029 | 1 | 1 | 0.037 | 1 | 1 | 1 | 1 |
| r=15 | 0.096 | 0.026 | 1 | 1 | 0.4 | 0.007 | 0.024 | 0.119 | 0.135 |
| r=20 | 1 | 1 | 1 | 1 | 1 | 1 | 1 | 1 | 1 |
| r=30 | 1 | 1 | 1 | 1 | 1 | 1 | 1 | 0.581 | 1 |
| r=40 | 1 | 1 | 1 | 1 | 1 | 1 | 1 | 0.37 | 0.219 |
| r=50 | 1 | 1 | 1 | 1 | 1 | 1 | 1 | 0.255 | 0.326 |
