# Supplementary material for: A Phylogenetic Perspective on the Individual Species-Area Relationship in Temperate and Tropical Tree Communities
Source: PLoS One. 2013 May 1;8(5):e63192. doi: 10.1371/journal.pone.0063192 (PMC3641141; doi:10.1371/journal.pone.0063192)
Supplement: Table S4 — The phylogenetic dispersion of species diversity accumulator at each scale of the nine forest dynamics plots based on NRI and NTI. (DOCX) [file pone.0063192.s008.docx]

**Table S4.** The phylogenetic dispersion of species diversity accumulator at each scale of the nine forest dynamics plots based on NRI and NTI.

| Radius  (m) | Ailao  shan | BCI | Edoro-1 | Edoro-2 | Korup | Lenda-1 | Lenda-2 | Wabikon Lake | Xishuangbanna |
| --- | --- | --- | --- | --- | --- | --- | --- | --- | --- |
| 1 | C | C | O | C | C | O | O | R | C |
| 2 | C | C | O | R | C | R | R | C | C |
| 3 | C | C | C | O | C | C | R | C | O |
| 4 | C | C | C | C | C | R | O | C | O |
| 5 | C | C | C | O | C | R | C | C | O |
| 6 | C | C | C | C | C | R | O | C | O |
| 7 | C | C | O | - | C | O | R | C | O |
| 8 | C | C | O | - | C | C | O | C | O |
| 9 | C | C | O | - | C | C | O | C | O |
| 10 | C | C | C | - | C | C | C | C | O |
| 11 | C | C | C | - | C | C | O | C | O |
| 12 | C | O | C | - | C | C | C | C | O |
| 13 | C | O | C | - | C | C | R | C | O |
| 14 | C | O | C | - | C | C | C | C | O |
| 15 | C | O | C | - | C | C | C | C | O |
| 16 | C | O | C | - | C | C | C | C | O |
| 17 | C | O | C | - | C | C | - | C | C |
| 18 | C | O | C | - | C | C | R | C | C |
| 19 | C | C | C | - | C | C | - | C | C |
| 20 | C | C | C | - | C | C | R | C | C |
| 21 | C | O | O | - | C | C | R | C | C |
| 22 | C | C | R | - | C | C | R | C | C |
| 23 | C | C | C | - | C | C | R | C | O |
| 24 | C | C | C | - | C | C | R | C | O |
| 25 | C | C | R | - | C | C | R | C | C |
| 26 | C | C | R | R | C | C | C | C | O |
| 27 | C | C | R | R | C | R | C | C | O |
| 28 | C | C | R | R | C | - | C | C | O |
| 29 | R | C | R | R | C | - | R | C | O |
| 30 | R | C | R | R | C | R | R | C | C |
| 31 | R | C | R | R | C | R | R | C | C |
| 32 | R | C | R | R | C | - | R | C | C |
| 33 | R | C | R | R | C | - | C | C | C |
| 34 | C | C | R | R | R | - | R | C | C |
| 35 | R | C | R | R | R | - | R | C | C |
| 36 | R | C | O | C | R | R | R | C | C |
| 37 | C | O | O | C | C | R | R | C | C |
| 38 | C | O | O | C | C | R | R | C | C |
| 39 | C | O | O | R | C | R | R | C | C |
| 40 | C | O | O | R | C | R | R | C | C |
| 41 | C | R | O | C | C | R | R | O | C |
| 42 | R | R | R | C | C | R | R | O | C |
| 43 | R | - | R | C | C | R | R | O | C |
| 44 | R | - | R | C | C | R | R | O | C |
| 45 | R | - | - | R | C | R | - | O | C |
| 46 | R | - | - | R | C | R | - | C | C |
| 47 | R | - | - | R | O | R | - | O | C |
| 48 | R | - | - | R | O | R | - | C | C |
| 49 | R | - | R | R | C | R | - | C | O |
| 50 | R | - | R | R | C | R | - | C | O |

C, R and O are three types of phylogenetic dispersion of group “species diversity accumulator”. C represents phylogenetic clustering, O represents phylogenetic overdispersion, R represents phylogenetic random and ‘-’ represent no species at this scale.
