# Supplementary material for: A Phylogenetic Perspective on the Individual Species-Area Relationship in Temperate and Tropical Tree Communities
Source: PLoS One. 2013 May 1;8(5):e63192. doi: 10.1371/journal.pone.0063192 (PMC3641141; doi:10.1371/journal.pone.0063192)
Supplement: Table S6 — The lineage information for the target species designated to be species accumulator or repeller on certain scales in the nine forest plots. This table does not include the target species designated to be accumulator on some scales and repeller on some other scales. (DOCX) [file pone.0063192.s010.docx]

**Table S6.** The lineage information for the target species designated to be species accumulator or repeller on certain scales in the nine forest plots. This table does not include the target species designated to be accumulator on some scales and repeller on some other scales.

| Plot | Species | Family | Order | Status | Scale (m) |
| --- | --- | --- | --- | --- | --- |
| Ailaoshan | *Cotoneaster dielsianus* var*. dielsianus* | Rosaceae | Rosales | Accumulator | 3-5, 11, 13 |
| Ailaoshan | *Ilex micrococca* | Aquifoliaceae | Aquifoliales | Accumulator | 4-5, 50 |
| Ailaoshan | *Lithocarpus hancei* | Fagaceae | Fagales | Repeller | 1-10 |
| Ailaoshan | *Vaccinium duclouxii* | Ericaceae | Ericales | Repeller | 2-18 |
| BCI | *Acacia melanoceras* | Fabaceae-mimosoideae | Fabales | Repeller | 1-10 |
| BCI | *Annona spraguei* | Annonaceae | Magnoliales | Accumulator | 3-27 |
| BCI | *Cecropia obtusifolia* | Urticaceae | Rosales | Accumulator | 1-27 |
| BCI | *Clidemia octona* | Melastomataceae | Myrtales | Accumulator | 9 |
| BCI | *Cupania rufescens* | Sapindaceae | Sapindales | Accumulator | 4-8 |
| BCI | *Elaeis oleifera* | Arecaceae | Arecales | Repeller | 2-10 |
| BCI | *Hampea appendiculata* | Malvaceae | Malvales | Accumulator | 3-25 |
| BCI | *Inga marginata* | Fabaceae-mimosoideae | Fabales | Accumulator | 1-27 |
| BCI | *Inga punctata* | Fabaceae-mimosoideae | Fabales | Accumulator | 7-13 |
| BCI | *Miconia argentea* | Melastomataceae | Myrtales | Accumulator | 1-37 |
| BCI | *Platymiscium pinnatum* | Fabaceae-papilionoideae | Fabales | Accumulator | 1-9 |
| BCI | *Psychotria graciliflora* | Rubiaceae | Gentianales | Accumulator | 3-7 |
| BCI | *Psychotria marginata* | Rubiaceae | Gentianales | Accumulator | 2-7 |
| BCI | *Rinorea sylvatica* | Violaceae | Malpighiales | Repeller | 1, 5-21 |
| BCI | *Sapium glandulosum* | Euphorbiaceae | Malpighiales | Accumulator | 3-25 |
| BCI | *Spondias radlkoferi* | Anacardiaceae | Sapindales | Accumulator | 1-33 |
| BCI | *Zanthoxylum ekmanii* | Rutaceae | Sapindales | Accumulator | 3-31 |
| BCI | *Zanthoxylum panamense* | Rutaceae | Sapindales | Accumulator | 2-35 |
| Edoro_1 | *Aidia micrantha* | Rubiaceae | Gentianales | Accumulator | 1-2 |
| Edoro_1 | *Barteria fistulosa* | Passifloraceae | Malpighiales | Repeller | 1-3 |
| Edoro_1 | *Barteria nigitiana* | Passifloraceae | Malpighiales | Repeller | 2 |
| Edoro_1 | *Cleistanthus michelsonii* | Euphorbiaceae | Malpighiales | Accumulator | 1 |
| Edoro_1 | *Dialium corbisieri* | Fabaceae-caesalpinioideae | Fabales | Accumulator | 1 |
| Edoro_1 | *Dialium pentandrum* | Fabaceae-caesalpinioideae | Fabales | Accumulator | 1, 4-5 |
| Edoro_1 | *Drypetes bipindensis* | Euphorbiaceae | Malpighiales | Accumulator | 1-4, 9-10 |
| Edoro_1 | *Drypetes bipindensis* | Euphorbiaceae | Malpighiales | Accumulator | 1-6 |
| Edoro_1 | *Drypetes capilipes* | Euphorbiaceae | Malpighiales | Repeller | 1 |
| Edoro_1 | *Drypetes ituriensis* | Euphorbiaceae | Malpighiales | Accumulator | 1 |
| Edoro_1 | *Englerophytum iturense* | Sapotaceae | Ericales | Accumulator | 1 |
| Edoro_1 | *Gillbertiodendron dewevrei* | Fabaceae-caesalpinioideae | Fabales | Repeller | 3-50 |
| Edoro_1 | *Greeneayodendron suaveolens* | Annonaceae | Magnoliales | Accumulator | 1 |
| Edoro_1 | *Guarea cedrata* | Meliaceae | Sapindales | Accumulator | 3 |
| Edoro_1 | *Julbernardia seretii* | Fabaceae | Fabales | Accumulator | 1-3 |
| Edoro_1 | *Lychnodiscus cerospermus* | Sapindaceae | Sapindales | Accumulator | 1 |
| Edoro_1 | *Pancovia harmsiana* | Sapindaceae | Sapindales | Accumulator | 1-21 |
| Edoro_1 | *Rothmannia longiflora* | Violaceae | Malpighiales | Accumulator | 1, 8-10 |
| Edoro_1 | *Scaphopetalum dewevrei* | Malvaceae | Malvaceae | Accumulator | 1-41 |
| Edoro_1 | *Uvariopsis congolana* | Annonaceae | Magnoliales | Accumulator | 1 |
| Edoro_1 | *Warneckea membranifolia* | Melastomataceae | Myrtales | Accumulator | 1-2 |
| Edoro-2 | *Anthonotha macrophylla* | Fabaceae-caesalpinioideae | Fabales | Repeller | 11-12 |
| Edoro-2 | *Barteria fistulosa* | Passifloraceae | Malpighiales | Repeller | 1-3 |
| Edoro-2 | *Leptonychia multiflora* | Malvaceae | Malvaceae | Accumulator | 3-6 |
| Edoro-2 | *Leptonychia zenkeri* | Malvaceae | Malvaceae | Accumulator | 3, 5 |
| Edoro-2 | *Lychnodiscus cerospermus* | Sapindaceae | Sapindales | Repeller | 1 |
| Edoro-2 | *Microdesmis puberula* | Pandaceae | Malpighiales | Accumulator | 1, 3-4 |
| Edoro-2 | *Strombosia pustulata* | Olacaceae | Santalales | Accumulator | 3 |
| Edoro-2 | *Strombosia tetrandra* | Olacaceae | Santalales | Accumulator | 5 |
| Edoro-2 | *Synsepalum stipulatum* | Sapotaceae | Ericales | Repeller | 3 |
| Edoro-2 | *Trichilia rubescens* | Meliaceae | Sapindales | Accumulator | 1 |
| Edoro-2 | *Warneckea membranifolia* | Melastomatceae | Myrtales | Accumulator | 3 |
| Korup | *Allexis caulifora* | Violaceae | Malpighiales | Repeller | 2 |
| Korup | *Baphia laurifolia* | Fabaceae-papilionoideae | Fabales | Accumulator | 1-6 |
| Korup | *Campylospermum calanthum* | Ochnaceae | Malpighiales | Accumulator | 1-12 |
| Korup | *Cola chlamydantha* | Malvaceae | Malvales | Accumulator | 3, 6-10 |
| Korup | *Cola digitata* | Malvaceae | Malvales | Accumulator | 1-22 |
| Korup | *Cola lateritia* | Malvaceae | Malvales | Accumulator | 4, 6-8 |
| Korup | *Cola lepidota* | Malvaceae | Malvales | Repeller | 2 |
| Korup | *Cola rostrata* | Malvaceae | Malvales | Accumulator | 1-18 |
| Korup | *Cola semecarpophylla* | Malvaceae | Malvales | Accumulator | 1-16 |
| Korup | *Crotonogyne strigosa* | Euphorbiaceae | Malpighiales | Accumulator | 1-24 |
| Korup | *Dasylepis blackii* | Salicaceae | Malpighiales | Accumulator | 1 |
| Korup | *Diospyros iturensis* | Ebenaceae | Ericales | Repeller | 2-5 |
| Korup | *Fagara macrophylla* | Rutaceae | Sapindales | Accumulator | 1-2, 4 |
| Korup | *Homalium sp.* | Salicaceae | Malpighiales | Repeller | 1-18 |
| Korup | *Jollydora duparquetiana* | Connaraceae | Oxalidales | Accumulator | 2-10 |
| Korup | *Leptonychia echinocarpa* | Malvaceae | Malvales | Repeller | 2-20 |
| Korup | *Leptonychia pallida* | Malvaceae | Malvales | Accumulator | 1-10 |
| Korup | *Mareyopsis longifolia* | Euphorbiaceae | Malpighiales | Accumulator | 1, 5, 8-10 |
| Korup | *Microcos coriacea* | Malvaceae | Malvales | Accumulator | 1 |
| Korup | *Ouratea sp.2* | Ochnaceae | Malpighiales | Accumulator | 2-5 |
| Korup | *Pentadesma butyracea* | Clusiaceae | Malpighiales | Accumulator | 2, 4-5 |
| Korup | *Phyllanthus sp.* | Euphorbiaceae | Malpighiales | Accumulator | 3 |
| Korup | *Phyllobotryon spathulatum* | Salicaceae | Malpighiales | Accumulator | 1 |
| Korup | *Protomegabaria stapfiana* | Euphorbiaceae | Malpighiales | Accumulator | 1-11 |
| Korup | *Pycnocoma macrophylla* | Euphorbiaceae | Malpighiales | Accumulator | 1-6 |
| Korup | *Rauvolfia vomitoria* | Apocynaceae | Gentianales | Repeller | 2, 5 |
| Korup | *Rinorea lepidobotrys* | Violaceae | Malpighiales | Accumulator | 1-20 |
| Korup | *Rinorea longicuspis* | Violaceae | Malpighiales | Accumulator | 2-18 |
| Korup | *Rinorea oblongifolia* | Violaceae | Malpighiales | Accumulator | 1-33 |
| Korup | *Rinorea sp.2* | Violaceae | Malpighiales | Accumulator | 1-26 |
| Korup | *Rinorea sp.3* | Violaceae | Malpighiales | Accumulator | 1-15 |
| Korup | *Strombosia scheffleri* | Olacaceae | Santalales | Repeller | 2, 5 |
| Korup | *Strombosiopsis tetrandra* | Olacaceae | Santalales | Repeller | 2-20 |
| Korup | *Synsepalum stipulatum* | Sapotaceae | Ericales | Repeller | 1-50 |
| Korup | *Turraeanthus mannii* | Meliaceae | Sapindales | Accumulator | 1-7 |
| Lenda_1 | *Alchornea floribunda* | Euphorbiaceae | Malpighiales | Repeller | 1-4 |
| Lenda_1 | *Celtis mildbraedii* | Ulmaceae | Rosales | Accumulator | 2-3 |
| Lenda_1 | *Desplatsia dewevrei* | Malvaceae | Malvales | Accumulator | 3 |
| Lenda_1 | *Diospyros bipindensis* | Ebenaceae | Ericales | Accumulator | 1, 7 |
| Lenda_1 | *Gilbertiodendron dewevrei* | Fabaceae-caesalpinioideae | Fabales | Repeller | 2-22 |
| Lenda_1 | *Julbernardia seretii* | Fabaceae | Fabales | Accumulator | 6-21 |
| Lenda_1 | *Leptonychia multiflora* | Malvaceae | Malvales | Accumulator | 9-30 |
| Lenda_1 | *Myrianthus preussii* | Urticaceae | Rosales | Accumulator | 9-19 |
| Lenda_1 | *Ouratea elongata* | Ochnaceae | Malpighiales | Accumulator | 8-13, 15, 18-26 |
| Lenda_1 | *Pancovia harmiana* | Sapindaceae | Sapindales | Accumulator | 1 |
| Lenda_2 | *Alchornea floribunda* | Euphorbiaceae | Malpighiales | Repeller | 1-8 |
| Lenda_2 | *Anthonotha macrophylla* | Fabaceae-caesalpinioideae | Fabales | Accumulator | 8-18 |
| Lenda_2 | *Diospyros bipindensis* | Ebenaceae | Ericales | Accumulator | 1-2, 8-12 |
| Lenda_2 | *Drypetes calvescens* | Euphorbiaceae | Malpighiales | Accumulator | 5, 9-10, 14-16 |
| Lenda_2 | *Gilbertiodendron dewevrei* | Fabaceae-caesalpinioideae | Fabales | Repeller | 2-21 |
| Lenda_2 | *Leptonychia multiflora* | Malvaceae | Malvales | Accumulator | 5 |
| Lenda_2 | *Pausinystalia macroceras* | Rubiaceae | Gentianales | Accumulator | 10 |
| Lenda_2 | *Pavetta nitidula* | Rubiaceae | Gentianales | Accumulator | 5 |
| Lenda_2 | *Staudtia kamerunensis* | Myristicaceae | Magnoliales | Repeller | 3, 6-12 |
| Lenda_2 | *Trichilia rubescens* | Meliaceae | Sapindales | Accumulator | 5-8 |
| Wabikon Lake | *Acer saccharum* | Sapindaceae | Sapindales | Accumulator | 5, 7-20 |
| Wabikon Lake | *Fraxinus americana* | Oleaceae | Lamiales | Repeller | 3, 4-6 |
| Xishuangbanna | *Aidia yunnanensis* | Rubiaceae | Gentianales | Accumulator | 1-25 |
| Xishuangbanna | *Albizia odoratissima* | Fabaceae-mimosoideae | Fabales | Repeller | 7, 9-10 |
| Xishuangbanna | *Alstonia rostrata* | Apocynaceae | Gentianales | Repeller | 22-31 |
| Xishuangbanna | *Amoora duodecimantha* | Meliaceae | Sapindales | Accumulator | 2-7 |
| Xishuangbanna | *Amoora yunnanensis* | Meliaceae | Sapindales | Accumulator | 3-17 |
| Xishuangbanna | *Antidesma japonicum* | Phyllanthaceae | Malpighiales | Accumulator | 1-27 |
| Xishuangbanna | *Antidesma montanum* | Phyllanthaceae | Malpighiales | Accumulator | 2-9 |
| Xishuangbanna | *Aporusa yunnanensis* | Phyllanthaceae | Malpighiales | Accumulator | 5 |
| Xishuangbanna | *Aquilaria yunnanensis* | Thymelaeaceae | Malvales | Accumulator | 5-37 |
| Xishuangbanna | *Ardisia thyrsiflora* | Myrsinaceae | Ericales | Accumulator | 23-32 |
| Xishuangbanna | *Beilschmiedia robusta* | Lauraceae | Laurales | Accumulator | 19-23 |
| Xishuangbanna | *Beilschmiedia roxburghiana* | Lauraceae | Laurales | Accumulator | 2-17 |
| Xishuangbanna | *Canarium subulatum* | Burseraceae | Sapindales | Accumulator | 1-7 |
| Xishuangbanna | *Castanopsis echidnocarpa* | Fagaceae | Fagales | Repeller | 9-50 |
| Xishuangbanna | *Castanopsis hystrix* | Fagaceae | Fagales | Repeller | 1, 14, 29-36 |
| Xishuangbanna | *Castanopsis indica* | Fagaceae | Fagales | Accumulator | 2-15 |
| Xishuangbanna | *Castanopsis megaphylla* | Fagaceae | Fagales | Accumulator | 1-29 |
| Xishuangbanna | *Chisocheton siamensis* | Meliaceae | Sapindales | Accumulator | 2-11 |
| Xishuangbanna | *Cinnamomum bejolghota* | Lauraceae | Laurales | Accumulator | 1-42 |
| Xishuangbanna | *Cleidion brevipetiolatum* | Euphorbiaceae | Malpighiales | Repeller | 9-25 |
| Xishuangbanna | *Croton kongensis* | Euphorbiaceae | Malpighiales | Accumulator | 3-6 |
| Xishuangbanna | *Cryptocarya acutifolia* | Lauraceae | Laurales | Accumulator | 2-7 |
| Xishuangbanna | *Dendrocnide sinuata* | Urticaceae | Rosales | Repeller | 2-3, 14-27 |
| Xishuangbanna | *Dichapetalum gelonioides* | Dichapetalaceae | Malpighiales | Accumulator | 2-47 |
| Xishuangbanna | *Diospyros hasseltii* | Ebenaceae | Ericales | Accumulator | 1-5 |
| Xishuangbanna | *Diospyros nigrocortex* | Ebenaceae | Ericales | Accumulator | 2-4 |
| Xishuangbanna | *Drypetes hoaensis* | Euphorbiaceae | Malpighiales | Accumulator | 1-13 |
| Xishuangbanna | *Duperrea pavettifolia* | Rubiaceae | Gentianales | Accumulator | 3-4 |
| Xishuangbanna | *Dysoxylum binectariferum* | Meliaceae | Sapindales | Accumulator | 2, 4-12 |
| Xishuangbanna | *Dysoxylum hongkongense* | Meliaceae | Sapindales | Accumulator | 2-3, 6, 8 |
| Xishuangbanna | *Engelhardia spicata* | Juglandaceae | Juglandales | Accumulator | 12-19 |
| Xishuangbanna | *Epiprinus siletianus* | Euphorbiaceae | Malpighiales | Accumulator | 1, 3-4 |
| Xishuangbanna | *Eurya austroyunnanensis* | Pentaphylacaceae | Ericales | Accumulator | 1-48 |
| Xishuangbanna | *Lasiococca comberi* var*. pseudoverticillata* | Euphorbiaceae | Malpighiales | Repeller | 1, 4-15 |
| Xishuangbanna | *Sumbaviopsis albicans* | Euphorbiaceae | Malpighiales | Repeller | 3-49 |
| Xishuangbanna | *Urophyllum chinense* | Rubiaceae | Gentianales | Accumulator | 3-4, 6, 17-36 |
